# *In Vivo* Electrochemical Analysis of a PEDOT/MWCNT Neural Electrode Coating

**DOI:** 10.3390/bios5040618

**Published:** 2015-10-13

**Authors:** Nicolas A. Alba, Zhanhong J. Du, Kasey A. Catt, Takashi D. Y. Kozai, X. Tracy Cui

**Affiliations:** 1 Department of Bioengineering, University of Pittsburgh, 5056 Biomedical Science Tower 3, 3501 Fifth Avenue, Pittsburgh, PA 15213, USA; E-Mails: nicolasaalba@gmail.com (N.A.A.); duzhanhong@gmail.com (Z.J.D.); kaseycatt@gmail.com (K.A.C.); tdk18@pitt.edu (T.D.K.); 2 Center for the Neural Basis of Cognition, University of Pittsburgh, Pittsburgh, PA 15260, USA; 3 McGowan Institute for Regenerative Medicine, University of Pittsburgh, Pittsburgh, PA 15260, USA; 4 NeuroTech Center of the University of Pittsburgh Brain Institute, Pittsburgh, PA 15260, USA

**Keywords:** interface, neural prosthesis, drug release, controlled drug release, electroactive polymer, nanocomposite

## Abstract

Neural electrodes hold tremendous potential for improving understanding of brain function and restoring lost neurological functions. Multi-walled carbon nanotube (MWCNT) and dexamethasone (Dex)-doped poly(3,4-ethylenedioxythiophene) (PEDOT) coatings have shown promise to improve chronic neural electrode performance. Here, we employ electrochemical techniques to characterize the coating *in vivo*. Coated and uncoated electrode arrays were implanted into rat visual cortex and subjected to daily cyclic voltammetry (CV) and electrochemical impedance spectroscopy (EIS) for 11 days. Coated electrodes experienced a significant decrease in 1 kHz impedance within the first two days of implantation followed by an increase between days 4 and 7. Equivalent circuit analysis showed that the impedance increase is the result of surface capacitance reduction, likely due to protein and cellular processes encapsulating the porous coating. Coating’s charge storage capacity remained consistently higher than uncoated electrodes, demonstrating its *in vivo* electrochemical stability. To decouple the PEDOT/MWCNT material property changes from the tissue response, *in vitro* characterization was conducted by soaking the coated electrodes in PBS for 11 days. Some coated electrodes exhibited steady impedance while others exhibiting large increases associated with large decreases in charge storage capacity suggesting delamination in PBS. This was not observed *in vivo*, as scanning electron microscopy of explants verified the integrity of the coating with no sign of delamination or cracking. Despite the impedance increase, coated electrodes successfully recorded neural activity throughout the implantation period.

## 1. Introduction

Neural prostheses have seen effective use in a variety of applications, including auditory prostheses, visual prostheses, brain-computer interface, and even opto-electrical applications [1,2,3,4,5]. Several examples employ arrays of penetrating microelectrodes that are implanted into cortex to record neural activity with single cell resolution [1,6,7,8]. When chronically implanted, these electrodes typically exhibit a large degree of variability of recording performance metrics such as impedance [9,10], single-unit yield [10,11,12,13,14], and signal-to-noise ratio [10,11,13,14]. This unreliable recording performance has become a principal obstacle against the more widespread clinical translation of intracortical electrodes. The large degree of variability and degradation of performance are thought to be a product of several biological and non-biological factors [6,10,14,15,16,17,18,19,20,21]. Principal among these factors is the degree of tissue inflammation elicited by electrode implantation and chronic presence [19]. Several interrelated inflammation mechanisms including the development of an encapsulating glial scar and the progressive degeneration and death of local neurons have been theorized to play important roles in recording quality deterioration [14,17,20,21,22,23,24,25,26,27] (see [19] for review).

In light of these observations, novel intracortical electrode design has largely focused on improving electrical characteristics [28], reducing tissue reactivity through changes to electrode geometry [23,29,30,31], flexibility [23,31,32,33,34,35,36,37,38], and surface properties [39], employing biomaterial strategies to promote tissue stability [23,40,41,42,43], and incorporating drug release systems to introduce anti-inflammatory agents [44,45,46]. While drug release systems intended for intracortical electrode integration have typically been limited to microfluidics or slow-release gels and coatings, systems utilizing conducting polymers have been explored due to their on-demand release capabilities [47,48,49]. On-demand release is additionally intriguing as it allows for the creation of synthetic synapses through the controlled release of neurotransmitters in a manner that mimics neural and glial signaling [50]. In addition to their capacity for controlled drug release, conducting polymers offer a combination of performance advantages to recording and stimulating electrodes, including reduced impedance and increased charge storage capacity [49,51,52,53,54,55].

Poly(3,4-ethylenedioxythiophene), or PEDOT, is an example conducting polymer applicable to neural prosthesis employed for *in vivo* on demand drug delivery. PEDOT has exhibited excellent electrochemical stability [51,56,57] and electrolyte compatibility [58], and has been integrated onto chronic intracortical recording electrodes in a number of studies [23,51,53,54,55,59,60,61,62,63,64,65]. PEDOT has been shown to significantly reduce probe impedance without substantially increasing site geometric surface area, and can be applied using electrochemical synthesis directly onto recording site surfaces [23,51,54,55,62]. PEDOT coatings allow for highly reversible charge injection, and significantly increase charge storage capacity compared to uncoated surfaces [52,53,60,66,67]. PEDOT coatings also exhibit good electrical stability after both repeated stimulation pulsation [52,56,67] and chronic warm PBS bath immersion [68]. The benefits above have proven translatable to *in vivo* application, and studies of chronically implanted PEDOT-coated electrodes show that the coated electrodes exhibited lower impedance and improved recording characteristics compared to uncoated controls [53,59,61,62]. PEDOT coatings have also been shown to elicit tissue reaction comparable to bare platinum following short term (two weeks) implantation [69].

Multi-walled or single-walled carbon nanotubes (MWCNTs or SWCNTs) may be incorporated into PEDOT coatings as a sole dopant or co-dopant to mechanically reinforce the polymer and prevent the spallation and cracking previously observed during chronic stimulation [64,70]. MWCNT-doped PEDOT films have been evaluated *in vivo* chronically in rat brain over 6 weeks, where they demonstrated excellent coating stability, reduced astrocyte activation, and increased local neuronal density compared to bare platinum implants, though recording ability was not evaluated [63]. In a more recent study, MWCNT-doped PEDOT coated Au recording electrodes demonstrated significant improvement in chronic recording performance compared to PEDOT:PSS-coated Au recording sites [65]. Additionally, MWCNTs have been found to act as “nanoreservoirs” when pre-loaded with drug and incorporated into conductive polymer coatings intended for controlled drug release [71]. We have previously shown that dexamethasone-loaded PEDOT/MWCNT-coated electrodes exhibited lower impedances following implantation into rat dorsal root ganglion and 14 days of *in vivo* stimulation (1 h per day on 10 of 14 study days, with 200 Hz, 20 μA, charge balanced biphasic pulses), along with reduced inflammation and improved neuronal survival around electrodes compared to those left uncoated [72]. Dexamethasone is an anti-inflammatory corticosteroid that has been found to attenuate tissue response to intracortical probes when introduced systemically [73] and by way of a slow-release coating locally [46].

The purpose of the work presented here is to build upon our previous study by performing a more in-depth analysis of the biological and electrochemical material changes that occur at the MWCNT-doped PEDOT-coated tissue/electrode interface *in vivo* within the sub-chronic period, using impedance spectroscopy and equivalent circuit modeling. Several earlier studies noted that while the 1 kHz impedance of PEDOT-coated electrodes was significantly lower than uncoated electrodes *in vitro* and at early time points *in vivo*, within a week of implantation into cortex coated electrode impedances raise rapidly, sometimes to the point of being statistically indistinguishable from impedances of uncoated controls [53,59,66]. While intracortical electrodes generally exhibit steadily increasing 1 kHz impedances during the initial week post-implantation due to inflammation and edema [9,12,18], the impedance changes exhibited by coated electrodes appear more abrupt and with distinctive complex features.

While 1 kHz impedance is commonly used to characterize electrodes, EIS allows for the measurement of changes to impedance magnitude and phase across an interface over a wide range of frequencies [3,9,18], and equivalent circuit modeling of the EIS data can shed light on the details of the contributing factors of the interface. The intent of this work is to thoroughly characterize electrochemical behavior of the coated electrode/tissue interface and understand the underlying mechanisms of the dynamic changes of impedance. The electrochemical properties of coated electrodes both sub-acutely implanted *in vivo* and immersed within a PBS bath *in vitro* were studied with CV and EIS. The neural recording performance before and after CV stimulations and the coating integrity after 11 days of implantation were examined.

## 2. Experimental Section

### 2.1. Carbon Nanotube Preparation

Multi-walled carbon nanotubes were purchased (OD 20–30 nm, ID 5–10 nm, length 10–30 µm, purity >95%, Cheap Tubes Inc., Brattleboro, VT, USA) and functionalized using a previously published method [71]. In summary, 200 mg of nanotubes were sonicated for two hours at ambient temperature in a bath consisting of 100 mL 1:3 ratio of concentrated HNO_3_ and H_2_SO_4_ (Sigma-Aldrich Co., St. Louis, MO, USA), carboxylating the nanotube surfaces. The solution was then stirred at ambient temperature for 12 h. Treated nanotubes were collected by decantation following ultracentrifugation (16,000 RPM at 15 °C for 40 min) and sonicated for 10 min in DI water (Milli-Q, Millipore Co., Billerica, MA, USA). Centrifugation was repeated until the pH of the supernatant solution was 6.0. Nanotubes were then collected and the remaining solvent was evaporated off in an oven at 60 °C.

### 2.2. In Vivo Array Preparation

Floating Microelectrode Arrays (FMAs, Microprobes for Life Science, Gaithersburg, MD, USA) were sterilized using an ethylene oxide gas sterilizer (AN 74i, Andersen Products, Inc., Haw River, NC, USA) after which they were transferred to a sterile environment. Initial quality-control impedance testing of all array sites was performed in a sterile PBS bath using a potentiostat (Autolab PGSTAT128N with FRA2 impedance spectroscopy module and Nova 1.8 control software, Metrohm USA, Riverview, FL, USA) with a platinum counter and Ag/AgCl reference (10 Hz–30 kHz, 10 mV RMS). If measured impedances differed substantially from manufacturer-reported values, the array was electrochemically cleaned (constant −2 V for 20 s). After cleaning, impedance was re-measured and cleaning repeated if necessary. Following testing, each array was immersed in a sterile polymerization solution prepared identically as that used in Kolarcik *et al.* [72], prepared as follows: prepared acid-functionalized MWCNTs and dexamethasone 21-phosphate disodium salt (Sigma-Aldrich) were dissolved into DI water at a concentration of 1 mg/mL and 20 mg/mL respectively, and sonicated for one hour. Post-sonication, 3,4-ethylenedioxythiophene (Sigma-Aldrich) was added to a concentration of 0.02 M and triturated until dissolved. Half of the array sites were coated using electropolymerization, where a potentiostat (FAS 1 Femtostat, Gamry Instruments, Warminster, PA, USA) with a platinum counter and Ag/AgCl reference was used to apply a constant 1.3 V (*vs.* Ag/AgCl) coating potential for 30 s. The array sites to be coated were selected using an alternating arrangement to prevent positional bias. After coating, the array was rinsed using DI water and impedance measurement was repeated in sterile PBS. Arrays were allowed to continue soaking in PBS for 30 min to remove adsorbed dexamethasone, given a final rinse using DI water, and were then stored dry in a sterile enclosure.

In addition, single microelectrodes (Pt/Ir alloy, 12 µm diameter, parylene-C insulated, 30 µm length exposed tip with ~380 µm^2^ area, Microprobes for Life Science) identical to those within FMAs implanted *in vivo* were used to characterize the coating morphology using scanning electron microscopy (SEM). SEM was conducted at The University of Pittsburgh’s Center for Biological Imaging on a field emission SEM (6335F, JEOL USA Inc., Peabody, MA, USA). Coating adhesion was evaluated by inserting and removing coated microelectrodes from Long Evans rat cortex *in vivo* or from an agarose gel using a micromanipulator (SM-11, Narishige USA, Inc., East Meadow, NY, USA). Coating integrity was evaluated using impedance spectroscopy and SEM. Agarose gel was prepared by heating a stirred 5 mg/mL agarose (Fisher Scientific, Waltham, MA, USA) solution to 85 °C until clear, at which point it was allowed to cool and set.

### 2.3. Surgical Implantation

Prepared FMAs were implanted unilaterally into the right primary visual cortex, monocular area (V1M) of three male Long Evans rats. Each animal was anesthetized under 3% isoflurane and mounted onto a stereotaxic frame (Narishige, East Meadow, NY, USA). The skull was exposed and a 3 × 3 mm craniotomy centered at 6.5 mm post Bregma and 3.5 mm lateral to midline was made over V1 using a high speed drill and fine rongeurs. Saline was applied continuously onto the skull to suppress heat. The dura was resected using fine Vannas scissors, and the brain surface was moistened using gelfoam while stereotaxic hardware was put into place. Insertion of the FMA array was performed using a vacuum tip mounted to a hand-driven micromanipulator (SM-11, Narishige, East Meadow, NY, USA). The craniotomy was sealed using a low-viscosity silicone [74] (Dow Corning, Midland, MI, USA). Four skull screws were mounted around the craniotomy and a headcap was applied using UV-cured dental cement (Pentron Clinical, Orange, CA, USA). 0.3 mg/kg buprenorphine was administered twice daily for three days as a post-operative analgesic. Animals were provided with soft water-based diet gel immediately after surgery, and food and water were provided *ad libidem* for the remainder of the experiment. All animal care and procedures were performed under the approval of the University of Pittsburgh Institutional Animal Care and Use Committee (Protocol 0806735) and in accordance with regulations specified by the division of laboratory animal resources.

### 2.4. In Vivo Evaluation Schedule

Immediately after implantation and daily thereafter, animals were lightly anesthetized using 1%–3% isoflurane and subjected to cyclic voltammetry (CV) and recording. All coated and uncoated array sites were subjected to an identical CV program each session. Before and after CV, both spontaneous and evoked neural activity were recorded and impedance was measured across the entire array. This protocol allowed all metrics to be measured immediately before and immediately after each CV session, and tracked daily for the duration of the experiment. Each component of the session is described in detail below.

#### 2.4.1. Cyclic Voltammetry

CV was performed using a PGSTAT128N potentiostat. Sequentially on each channel, CV was performed using 20 cycles from −0.9 V to 0.6 V (*vs.* Pt. counter electrode) at a 1 V/s scan rate, anode-first. Redox behavior of each site was qualitatively observed in terms of reduction and oxidation peak height and potential shift. Charge storage capacity and charge balance were computed by integrating the area under cathodic and anodic curves, and cathodic and anodic charge capacities were summed to yield the total charge storage capacity of each electrode.

#### 2.4.2. Neurophysiological Recording

Recording of spontaneous and visually evoked single units, multi-unit, and LFP response was performed each session using previously established methods [13], both before and after CV. During each recording session, animals were situated on a heating pad inside of a darkened faraday cage (1.6 mm mesh) while lightly anesthetized with isoflurane. An LCD screen was positioned outside of the cage and the animal’s head was fixed to provide for optimum viewing angle from the dominant eye. Optimum activity was typically observed when isoflurane levels were set at the very lowest concentration sufficient for the maintenance of sedation (1.5%–1.75%). Spontaneous recording was conducted in a dark room. Visual stimuli was presented using the MATLAB-based Psychophysics toolbox [75,76,77] on an LCD monitor placed 20 cm from the eye contralateral to the implant. Solid black and white bar gratings were presented drifting in a perpendicular direction and synchronized with the recording system (RX5, Tucker-Davis Technologies, Alachua, FL, USA). Each 4 s grating presentation (rotated in 45° increments) was separated by a 4 s dark screen period. Additionally, a spiraling continuous stimulation with 3°/s clockwise rotation was also presented each recording session. The raw data stream was filtered to produce LFP (1–300 Hz) and spike (0.3–5 kHz) data streams. The spike data stream was further pre-processed using published methods [78,79]. Possible spikes were detected using a fixed negative threshold value of 3.5 SD. Offline spike sorting was carried out using a custom MATLAB script modified from previously published methods [80,81]. Average signal-to-noise ratio (averaging the amplitudes of single units for each channel) and average amplitude of noise (4 SD) were used to quantify electrode recording performance. Only channels exhibiting detected spikes were included in SNR computation.

#### 2.4.3. Impedance Spectroscopy and Equivalent Circuit Analysis

Electrochemical impedance spectra were measured before and after each CV session. While under anesthesia, the implanted array was connected to the Autolab potentiostat using a 16 channel multiplexer. Impedance was measured for each channel using a 10 mV RMS sine wave from 10 Hz to 32 kHz, employing a 15 multisine paradigm to shorten the time required for measurement. MEISP (v3.0, Kumho, Seoul, Korea) and NOVA (v1.8, Metrohm USA) were used for measurement and analyses.

### 2.5. Explant Imaging

Coating integrity of the explanted probes was evaluated using scanning electron microscopy. Following array extraction, electrodes were soaked in a 5% trypsin solution for twenty minutes at ambient temperature to remove tissue residue and reveal the underlying coating surface. Arrays were then rinsed with DI water and dried for high resolution SEM.

### 2.6. In Vitro Coating Evaluation

In order to decouple the underlying mechanisms behind the sub-acute changes to *in vivo* electrochemical behavior from the material property changes of the coating, PEDOT/MWCNT/Dex coatings were evaluated *in vitro* in a controlled environment. A custom array was ordered from Microprobes for Life Science featuring ten 4 cm long platinum/iridium microelectrodes with composition and tip geometry identical to those implanted *in vivo*. Each electrode shank featured an additional polyimide sleeve to minimize capacitive shunting, in addition to the parylene-C insulation. Electrode tips were coated with MWCNT and dexamethasone-doped PEDOT in a manner identical to that described in Section 2.2. The impedance of each electrode was measured before and after coating to ensure proper electrochemical behavior and coating application. A custom chamber was constructed by drilling a small hole in the cap of a 15 mL polypropylene centrifuge tube (Becton Dickenson and Company, Franklin Lakes, NJ, USA) after which the array was secured in place within the cap using clay to make an airtight seal. The chamber was filled with 12 mL of sterile PBS and sealed by screwing on the tube cap with the array fixed in place. Once a day for eleven days, five of the array electrodes were subjected to CV and charge storage capacity measurement as in Section 2.4.1, with impedance measured before and after CV as in Section 2.4.3. The other five array electrodes were subjected to daily impedance measurement without CV. Throughout the eleven days of testing, the chamber was sealed at ambient temperature and the electrode tips were continuously immersed.

### 2.7. Statistics

Comparison between two groups was performed using a two-tailed Welch’s *t*-test. Comparison between multiple groups was performed using one-way standard ANOVA with a Tamhane T2 post-hoc test. Tamhane T2 was selected in place of Tukey due to the large difference in variances within *in vivo* impedance data. For all tests, α < 0.01 indicated a significant result.

## 3. Results

### 3.1. Pre-Implantation Coating Characterization

**Figure 1:**
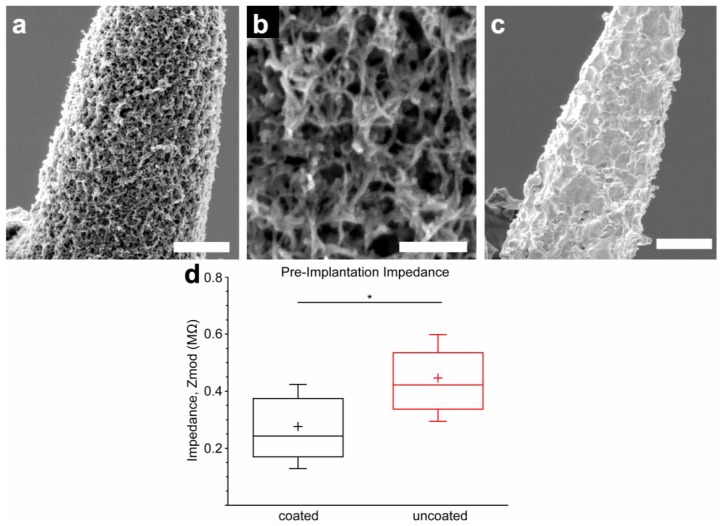
(**a**) SEM image of PEDOT/MWCNT/Dex-coated Pt/Ir microelectrode (scale bar = 3 µm); (**b**) Magnified image of (**a**), revealing an open, nanofibrous morphology (scale bar = 0.5 µm); (**c**) SEM image of uncoated Pt/Ir microelectrode (scale bar = 3 µm) shows roughness due to arc plasma fabrication method; (**d**) Coated electrodes have significantly lower 1 kHz impedance than uncoated electrodes before implantation, due to the increased surface area provided by the coating morphology (*N* = 24, box = 25%–75%, cross = mean, whiskers = SD). * *p* < 0.01.

Dexamethasone (Dex) and MWCNT-doped PEDOT coatings were characterized with regard to morphology and impedance (Figure 1). Representative images are shown in Figure 1a,b, demonstrating a uniform nanofibrous, open, lattice-like morphology of the film. This is in contrast to uncoated microwires, which exhibit the coarse and irregular texture typical of arc-exposed electrode tips (Figure 1c). The contrast demonstrated in the scanning electromicrographs illustrates the greatly increased surface area of the coated surfaces. The impact of this increased surface area was observed using impedance measurement (Figure 1d), which demonstrated that the coating decreased to a statistically significant degree (*p* = 0.0003) the 1 kHz impedance modulus of the coated microwire tips (276 kΩ ± 147 kΩ) compared to those left uncoated (446 kΩ ± 153 kΩ) in PBS. Coating adhesion testing demonstrated no apparent changes to electrode impedance or surface morphology following insertion and removal of a coated microwire from *in vivo* rat cortex. Insertion and removal of a coated microwire from agarose gel resulted in a clinging residue of agarose to the surface visible by SEM, but no change in electrode impedance.

### 3.2. Electrochemical Impedance in Vivo

**Figure 2:**
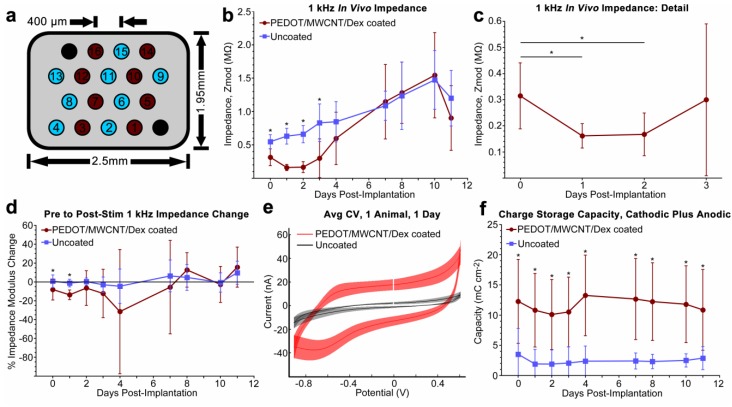
(**a**) FMA layout of uncoated Pt/Ir electrodes (light blue), PEDOT/MWCNT/Dex-coated (dark red), and ground and reference electrodes (black); (**b**) Average 1 kHz *in vivo* impedance, recorded before release stimulus. Significant differences were observed over the initial three days, then disappeared suggesting that the benefit either was negated (*N* = 24 for days 0–3, 16 for days 4–11); (**c**) Coated probes in (**b**) exhibited an impedance reduction during the initial two days *in vivo*, possibly due to an electrolyte permeation process through the pores of the coating (*N* = 24); (**d**) Average impedance change from release stimulus (post-stim/pre-stim). After day 1, there was no difference in post-stim impedance change; (**e**) Example averaged CV stimulus curve from one animal at one day post-implantation. Discontinuity indicates starting potential. A reduction peak is apparent at ~−700 mV within the coated trace, indicating reduction and dopant release. The uncoated trace exhibits no peak; (**f**) Average total (anodic + cathodic) charge storage capacity. Capacity of coated electrodes remained ~300% greater for the implant duration, demonstrating coating stability. For all plots, gaps at day 5, 6, and 9 are due to potentiostat malfunction that resulted in loss of data. All data presented as mean ± SD. * *p* < 0.01.

To compare the *in vivo* performance of PEDOT/MWCNT/Dex-coated probes against conventional non-coated microwires, Long Evans rats were implanted with 16-channel floating microwire arrays unilaterally into V1 monocular cortex. The layout of the implanted arrays is illustrated (Figure 2a). Comparisons between sub-acute *in vivo* impedance and charge storage behavior were quantified (Figure 2). For all impedance and cyclic voltammetry measures, *N* = 24 for days 0–3, but was reduced to *N* = 16 for days 4–11, as a result of animal loss due to pneumonia. Day 0 data were collected on the same day as implantation, immediately after surgery. Data for days 5, 6, and 9 are not displayed, as potentiostat failure during script execution prevented pre-CV data collection from at least one animal.

Average daily pre-CV 1 kHz impedance modulus values for coated and uncoated probes are shown (Figure 2b). Impedances of the coated probes were found to be significantly lower than values observed from uncoated probes for the first three days post-implantation (*p* < 0.0001 for each day). Subsequently, the impedances of coated probes increased rapidly to the point that they on average became indistinguishable from uncoated values for the remainder of the experiment. Dynamic impedance behavior over the first three days of the experiment is shown in detail (Figure 2c), which highlights that coated probe impedance values remained significantly depressed compared to day 0 values for two days post-implantation (*p* = 0.004 for day 0–1, *p* = 0.007 for days 0–2), while uncoated probes on average exhibited steadily increasing impedance values. Daily values for the average change in probe 1 kHz impedance measured immediately before CV were compared to those measured immediately after CV (Figure 2d). Average post-CV impedance values typically changed by a degree less than 20% of pre-CV values, with change usually trending in the negative direction. Additionally, a statistically significant difference between coated and uncoated post-CV 1 kHz impedance change was only observed on days 0 and 1 (*p* = 0.002 and 0.0001, respectively).

### 3.3. Cyclic Voltammetry and Charge Storage Capacity in Vivo

Electrochemical properties of the deposited films were evaluated using cyclic voltammetry. 20 cycles between −0.9 V to 0.6 V at 1 V/s were applied to each channel daily and the resulting curves were used to characterize the sub-acute stability and charge capacity of the films *in vivo*. Typical *in vivo* CV curves are displayed (Figure 2e) for both coated and uncoated probes, with all channels from each group averaged from one animal and one day (day 1 post-implantation) and plotted within the same figure. Coated probes exhibited a pronounced reduction peak between −600 to −800 mV each day. Uncoated probes exhibited no visible redox behavior.

Average daily values of total charge storage capacity (*CSC_T_*) are shown for both coated and uncoated probes (Figure 2f), as a function of uncoated electrode surface area. As expected from the curves shown in Figure 2e, average *CSC_T_* of coated probes remained 490% ± 77% greater than uncoated probes for the duration of the experiment. The difference was found to be statistically significant at each time point (*p* < 0.01 for all). The average anodic-to-cathodic charge balance ratio of all coated electrodes across all days was 1.32 ± 0.22 (*n* = 176), indicating a degree of anodic charge buildup. The average percent change in *CSC_T_* of coated electrodes between day 1 and day 11 was found to be 4.6% ± 21.0% (*n* = 16), indicating only a small change of charge storage capacity over the course of the experiment, with no definite trend upwards or downwards.

### 3.4. Equivalent Circuit Modeling

**Figure 3:**
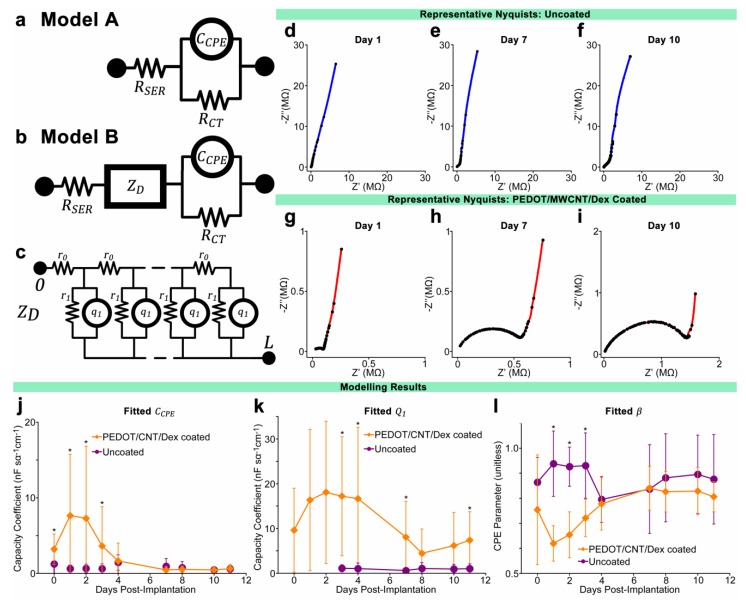
(**a**,**b**) Equivalent circuit models for uncoated electrodes in PBS or initial days post-implantation (model A) and uncoated electrodes at later days post-implantation as well as PEDOT/MWCNT/Dex-coated electrodes (model B), with distributed element ZD representing the polymer coating as well as tissue encapsulation; (**c**) Schematic of the dual-channel distributed diffusion impedance element ZD; (**d**–**i**) Representative Nyquist impedance plots of uncoated (**d**–**f**) and coated (**g**–**i**) electrodes at three time points post-implantation, with all plots exhibiting an identical frequency range (100 Hz to 33 kHz). Note contrast between uncoated and coated Nyquist plots at days 7 and 10 despite statistically identical 1 kHz impedance. The growing semi-circular features at higher frequencies within (**g**–**i**) suggest the development of encapsulation over coated electrodes not present in uncoated electrodes; (**j**–**l**) Average fitted values of modeling parameters CCPE, Q1, and β. Q1 were not fitted for days 0–2 for uncoated probes due to use of model A. Comparatively large values of CCPE during the initial four days indicates a coating benefit to electrode capacitance that diminished at later points. Reducing values of Q1 indicate a reduction in coating/electrolyte capacitance, possibly due to changing surface area. β exhibited low values during first four days, but later increased to values equivalent to uncoated electrodes, suggesting a change to the nature of the coating/electrode interface. *N* varied from session to session. All data presented as mean ± SD. * *p* < 0.01.

Curve fitting and equivalent circuit analysis was applied to the measured data using a method developed by Bisquert [82,83] that has since been employed to model the electrical characteristics of both *in vivo* inflammatory tissue encapsulation [84] as well as PPy/CNT films on intracortical electrodes *in vitro* [63,85]. Using this method, data is fitted to one of two models. Model A (Figure 3a) is a simple Randles circuit employing a constant phase element (CPE) in parallel with a resistor, and has been commonly used to model bare microelectrodes in electrolyte [86,87]. In this model, the CPE is representative of the double layer capacitance of the metal recording surface, while the parallel resistance *R_CT_* is representative of the charge transfer resistance, or the resistance of the material to the transfer of faradaic current. The CPE is modeled using two terms: *C_CPE_*, the coefficient of CPE capacitance per unit length (F·s^α−1^·cm^−1^), and β, a parameter defined by the phase angle of the CPE. β has a value between 0 and 1, where β = 1 represents an ideal capacitor with phase angle 90° and β = 0.5 represents a CPE with phase angle 45° (also known as a Warburg impedance). The physical correlate of β is not well understood, and is thought to be related to surface roughness, charge uniformity, coating bulk properties, or varying reaction rates along the electrode surface [87]. A second resistive element *R_SER_* represents the solution resistance of the bulk saline/tissue environment. For typical microelectrodes composed of a blocking material such as platinum, *R_CT_* is expected to be extremely high, leading to the electrode behavior being dominated by the probe capacitance [85]. In this study, model A was used to fit data collected from uncoated electrodes *in vitro* or at very early time points *in vivo,* in plots where multiple time-constant behavior was not observed.

Model B (Figure 3b) is similar to model A, only with an additional diffusion impedance element *Z_D_*, a double-channel transmission line distributed element representing a superposition between a solid and a liquid continuum, extending from the electrode surface with thickness *L*. A schematic representation of *Z_D_* is provided (Figure 3c), where *r*_0_ is the resistance per unit length (Ω·cm^−1^) of the electrolyte fluid, *r*_1_ is the charge transfer resistance length (Ω·cm) of the electrolyte/solid interface, and *q*_1_ is the coefficient of the interface CPE per unit length (F·s^α−1^·cm^−1^). An extensive mathematical treatment of this element can be found in the literature [82,84]. In summary, the diffusion resistance *Z_D_* is generally expressed as

(1) $$Z_{D}={\left\{\frac{R_{0}R_{1}}{\left[1+{\left(\frac{i\omega }{\omega_{1}}\right)}^{\alpha }\right]}\right\}}^{\frac{1}{2}}\times \coth\left\{{\left(\frac{\omega_{1}}{\omega_{L}}\right)}^{\frac{\alpha }{2}}{\left[1+{\left(\frac{i\omega }{\omega_{1}}\right)}^{\alpha }\right]}^{\frac{1}{2}}\right\}$$

(2) $$\omega_{L}={\left(r_{0}q_{1}L^{2}\right)}^{-\frac{1}{\alpha }}={\left(R_{0}Q_{1}\right)}^{-\frac{1}{\alpha }}$$

(3) $$\omega_{1}={\left(r_{1}q_{1}\right)}^{-\frac{1}{\alpha }}={\left(R_{1}Q_{1}\right)}^{-\frac{1}{\alpha }}$$

where *i* = $\sqrt{-1}$ and ω is the angular frequency (rad·s^−1^). If *L* is equated to 1 for fitting purposes, *R*_0_ becomes the total resistance of the electrolyte phase and represents restriction to ionic motion within the pores of the coating, while *R*_1_ and *Q*_1_ represent the total charge transfer resistance and CPE capacitance of the electrolyte/conducting polymer boundary with α representing the CPE exponential parameter, analogous to β above. An important stipulation of this model is that the resistivity of the solid phase must remain negligible, requiring the assumption that the PEDOT coating remains oxidized and highly conductive for the duration of the experiment [82]. This assumption can be justified due to the consistent values of *CSC_T_*, which suggest intact conduction between the coating and the metal electrode.

Representative Nyquist plots of recorded impedance data are shown (Figure 3d–i), with all plots exhibiting an identical frequency range (100 Hz to 33 kHz). Figure 3d–f display Nyquist plots of a representative uncoated electrode at days 1, 7, and 10, respectively, and Figure 3g–i display plots from a representative coated electrode at the same time points. When comparing plots, note that each plot is scaled to allow optimal viewing of the entire range of frequencies.

Model parameters were fitted to experimental data using a complex linear least squares fitting program. Before fitting, the thirteen lowest frequency impedance measurements from each impedance spectrum were removed to eliminate scatter due to low-frequency noise, which was found to be a consequence of the multisine measurement method. Also, impedance spectra that were found to contain enough broadband noise or measurement artifact to interfere with consistent fitting were removed.

Multiple trends were observed in the fitted model parameters, as shown in Figure 3j–l. Confidence in the modeled values is to a large part determined by the range of frequencies available for fitting, which in this study was limited to minimize the time required for measurement due to animal safety concerns. Parameters that are not well represented within the measured frequency range may vary substantially without changing the quality of the overall fit. The parameter representing platinum charge transfer resistance, *R_CT_*, is an example, as it is most relevant to frequencies much lower than those measured here (*f* < 1 Hz). Parameters demonstrating the most dynamic and consistent behavior in coated electrodes were found to be *C_CPE_* and *Q*_1_, representing the metal substrate and the coating or tissue encapsulation surface capacitance coefficients respectively. Their behavior compared to the same parameters modeled from uncoated electrode data is shown in Figure 3j,k. The CPE phase angle parameter of *C_CPE_* (β) also demonstrated dynamic change in the coated electrodes but remained at consistent elevated values in uncoated electrodes, as shown in Figure 3l, while the CPE parameter of *Q*_1_ (α) maintained a high value of between 0.85 and 1 for the duration of the experiment for both coated and uncoated electrodes. Pore resistance *R*_0_ and conducting polymer charge transfer resistance *R*_1_ of coated electrodes demonstrated a small degree of variation over time that did not correlate with impedance. The solution resistance *R_SER_* was found to be inconsequentially small compared to other elements and did not contribute substantially to quality of fits when varied manually.

### 3.5. Neurophysiological Recording

**Figure 4:**
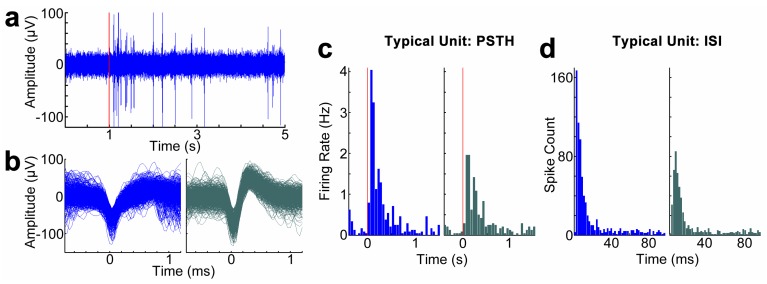

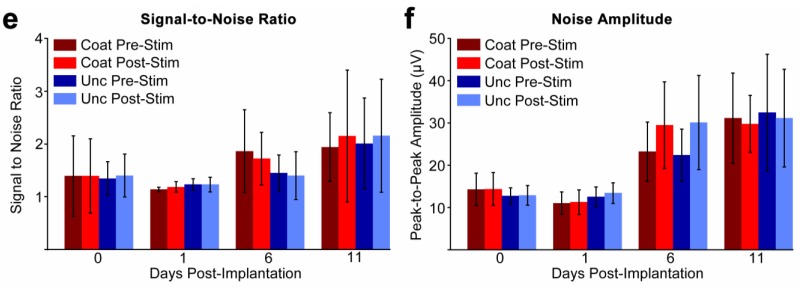
(**a**–**d**) Representative unit recording from a PEDOT/MWCNT/Dex-coated electrode at day 11 post-implantation. Red line indicates onset of visual stimulus. (**a**) Filtered (300 Hz–3 kHz) data stream; (**b**) Two example units sorted from the same coated electrode; (**c**) Peristimulus time histogram (PSTH) for each respective unit.; (**d**) Interspike interval (ISI) histogram of the two example units; (**e**) Average signal-to-noise ratio (SNR) values on representative days pre and post-release stimulus; (**f**) Average noise amplitude on representative days pre and post-release stimulus. All groups exhibited similar values within each day, suggesting equivalent performance. All data presented as mean ± SD. *p* > 0.01 for all.

Neurophysiological recording capability of coated electrodes compared to uncoated electrodes was evaluated through the visual stimulation of the array-implanted animals. Visual stimulation evoked robust firing rate change during the entire period of experimentation. A representative filtered (0.3–5 kHz) spike data stream from a coated channel on the last day of implantation is shown in Figure 4a, with visual stimulation initiation time indicated. Waveforms, inter-spike interval histograms, and PSTHs of two representative sorted single units on this channel are presented in Figure 4b–d. Average recording SNR (signal-to-noise ratio) and noise amplitude between the coated and uncoated electrodes are compared in Figure 4e,f. Same-day unit information is divided into groups before and after CV to evaluate the influence of CV on neural activity. Only channels exhibiting detectable spiking behavior were included in SNR computations, resulting in values of N between 8 and 15 per group. No significant difference was observed between pre and post-CV values of SNR or noise amplitude of either coated or uncoated electrodes at any time point (*p* > 0.01 for all). Performance was also not observed to be correlated with impedance during the initial week, as uncoated and coated probes exhibited the same noise amplitude and SNR despite having significantly different 1 kHz impedance. In general, the coated channels performed similarly in comparison with uncoated channels.

### 3.6. Explant Imaging

**Figure 5:**
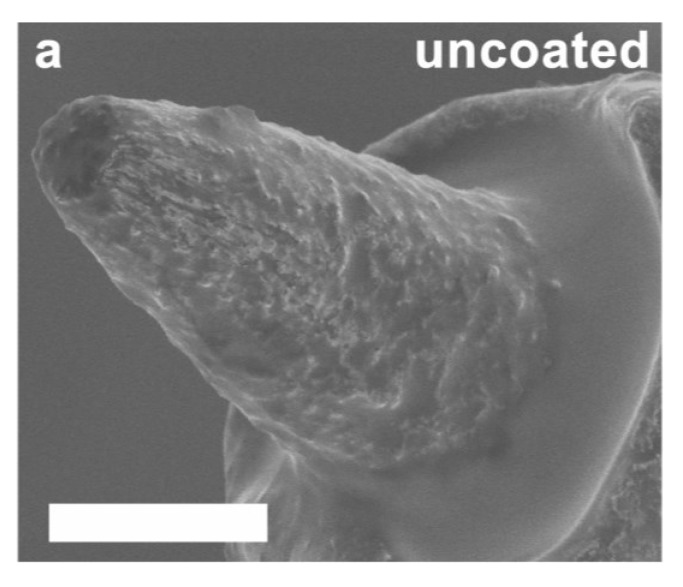

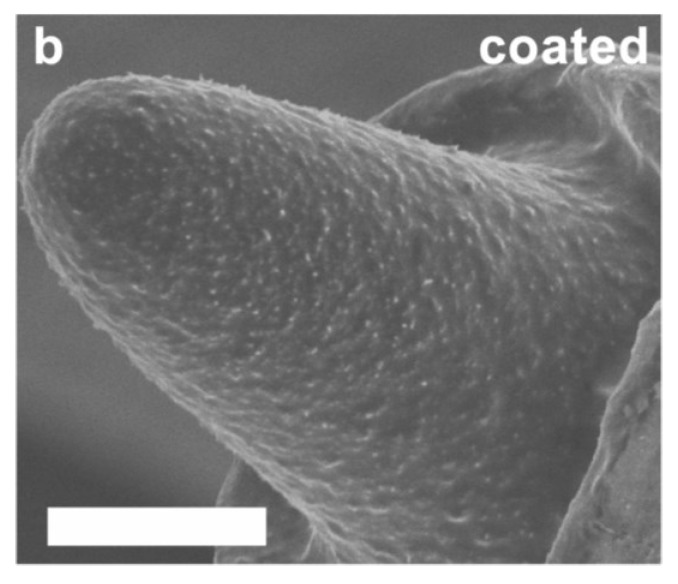
SEM images of representative uncoated (**a**) and PEDOT/MWCNT/Dex coated (**b**) electrode tips extracted from the brain after 11 days. Tips were cleaned using trypsinization and dried before imaging. Note intact coating with no visible cracks or spallation, and the presence of a dense biological film overlaying the coating. Scale bars = 3 µm.

Scanning electron microscope images of representative explanted electrodes are shown in Figure 5, including uncoated (Figure 5a) and coated (Figure 5b) examples. Uncoated explanted electrodes demonstrated dimensions and surface texture visually consistent with pre-implant micrographs. Coated explanted electrodes exhibited intact coatings with no visible cracks, spallation, or removal in over 85% of the electrodes examined. Tissue ingrowth was also observed on the surface of the intact coated explanted electrodes, penetrating and occluding the open lattice structure of the coating. We were unable to determine the composition of this residue due to the preparatory steps performed for high resolution SEM.

### 3.7. In Vitro Coating Evaluation

**Figure 6:**
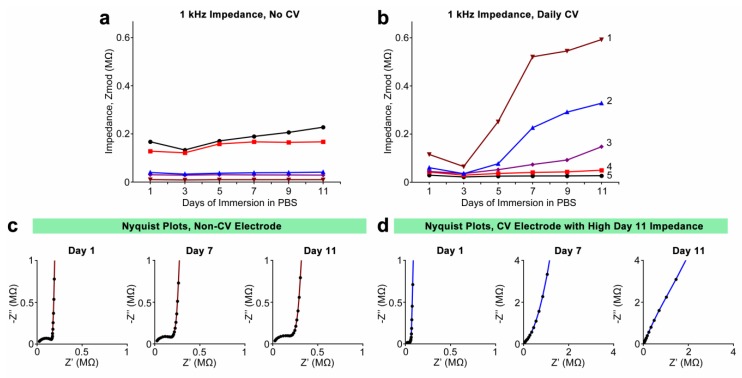
(**a**,**b**) 1 kHz impedances of individual PEDOT/MWCNT/Dex-coated electrodes chronically submerged in sterile PBS over 11 days. Electrodes in (**a**) were not subjected to daily CV, while those in (**b**) were subjected to daily CV. Electrodes in (**b**) are numerically labeled for reference; (**c**,**d**) Representative Nyquist plots collected at days 1, 7, and 11 for a non-CV-stimulated electrode (**c**) and a CV-stimulated electrode that exhibited high day-11 impedance ((**d**), Electrode 2). Note that non-CV-stimulated electrodes exhibited characteristic high-frequency behavior at all days, while high-impedance CV-stimulated electrodes exhibited a progressive “straightening” and loss of characteristic high-frequency behavior.

Daily 1 kHz impedance values for individual electrodes are shown in Figure 6a,b, with Figure 6a featuring electrodes only subjected to daily impedance measurement without CV, and Figure 6b featuring electrodes subjected to both daily impedance measurement and CV. Initial 1 kHz impedance values of coated electrodes ranged between 10 and 168 kOhm. As with the impedances of the electrodes implanted *in vivo*, this disparity can be attributed to variations in exposed tip surface area and coating thickness. Among the electrodes that were not subjected to CV, impedances were seen to remain consistent throughout the experiment, changing at most 35% between day 1 and 11 (Figure 6a). Those electrodes that were subjected to daily CV exhibited less consistent behavior. The two CV-stimulated electrodes with the lowest initial impedance behaved much like the non-CV-stimulated electrodes in Figure 6a, with very little change in impedance over the course of the experiment. However, three of the electrodes exhibited sharp increases in impedance between days 5 and 7, and by day 11 had increased between 230% and 440% from day 1 values (Figure 6b, with electrodes numbered for reference). CV of the electrodes in Figure 6b provided total charge storage capacity (*CSC_T_*) information. Initial *CSC_T_* values for these electrodes ranged between 5.8 and 38.0 mC·cm^−2^. The three electrodes that exhibited the greatest increases in 1 kHz impedance, electrodes 1, 2, and 3, also exhibited large decreases in *CSC_T_*, at 54%, 52%, and 48%, respectively. The electrodes that exhibited smaller impedance increases, electrodes 4 and 5, showed less charge storage capacity loss, at 16% and 37%, respectively. It should be noted that while electrode 5 exhibited the smallest impedance increase, it also exhibited the greater degree of *CSC_T_* reduction at 37% and also had the highest initial *CSC_T_*. Nyquist plots of a non-CV stimulated electrode (Figure 6c) and a CV-stimulated electrode (electrode 4) (Figure 6d) are shown at three different days, showing the evolution of complex impedance in each representative case. Plots in Figure 6c exhibit the bimodal frequency response characteristic of two distinct time constants, suggesting the presence of the coating at all days. Meanwhile, plots in Figure 6d exhibit a progressive “straightening” of the frequency response and the vanishing of the higher-frequency features, with the plot at day 11 suggesting only a single time constant consistent with a bare metal electrode. This phenomenon is present in the Nyquist plots of those other electrodes that exhibited large 1 kHz impedance increases.

## 4. Discussion

The objective of this study was to perform an in-depth electrochemical analysis of changes to Dex-loaded MWCNT-doped PEDOT coatings on platinum/iridium microwire electrodes following CV stimulation, both *in vivo* and *in vitro*. The drug release capability, tissue reactivity, and impedance characteristics of this coating within rat dorsal root ganglion was previously documented by our laboratory in Kolarcik *et al.* [72]. This current work extends the PNS neurostimulation application to the CNS visual stimulus-evoked neurophysiological recording, charge capacity evaluation, and a more comprehensive study of sub-acute *in vivo* electrode material property changes in the brain through equivalent circuit analysis. The early sub-acute phase post-implantation presents a critical time period for study as it is during the initial one to two weeks that the most dynamic electrical behavior is often observed, as well as the most extensive changes to the tissue inflammatory state. This is particularly true in the case of coated electrodes as it is during this initial period when surface fouling, pore clogging, and delamination are likely to occur.

Impedances of the coated probes remained within a range comparable to pre-implanted PBS measurements during the first two days of the experiment. However, the impedance values increased rapidly after three or four days *in vivo* and were characterized by distinct high-frequency reactance behavior in Nyquist plots, suggesting a bulk change in capacitance. Despite this capacitance change the recording performance of the coated electrodes did not diminish during the implantation period. To better understand the PEDOT material property component of this change in electrical properties, identical PEDOT/CNT/Dex-coated electrodes were characterized with EIS for the same duration in PBS. Half of these electrodes were also subjected to daily CV stimulus. Coatings subjected to the stimulus showed 1 kHz impedance increases similar to electrodes implanted *in vivo* over the same time scale. However, the coatings exhibited different charge storage capacity behavior and impedance frequency response *in vitro* compared to those *in vivo*. This suggests that the increases in 1 kHz impedance are the result of different mechanisms, such as coating delamination *in vitro* compared to encapsulation *in vivo*.

### 4.1. Deposition, Morphology, and In Vitro Electrochemical Properties

We found that electrodeposition using constant potential produced the most consistent PEDOT/MWCNT/Dex coatings with the greatest degree of impedance reduction on both gold macroelectrodes and Pt/Ir microelectrodes. This is in contrast with observations by Zhou *et al.* [63] who found that constant current polymerization resulted in better PEDOT/MWCNT films. Interestingly, our method produced the open nanofibrous lattice-like morphology exhibited in Figure 1a,b, which is similar to the PEDOT/CNT films without Dex that we previously reported [64,65], while films produced by Zhou *et al.* exhibited a more cauliflower-like morphology. A possible explanation is that our CNT size range and functionalization method may have resulted in a greater fraction of entrapped nanotubes, or a different rate of PEDOT deposition. Parameters were optimized for each type of electrode to provide the most similar impedance and morphology. A relatively high 1.3 V (*vs.* Ag/AgCl) was employed to coat Pt/Ir microelectrodes as lower potentials resulted in inconsistent impedances. Deposition on Pt/Ir was carried out to a charge density of approximately 0.29 C/cm^2^. Careful MWCNT functionalization and suspension preparation were critical to achieving consistent and robust coatings. Insufficient MWCNT carboxylation was found to result in clumping of the nanotubes in solution, poor dispersion, and poor, non-uniform integration into the coating.

SEM imaging of coatings revealed an open and porous morphology (Figure 1a,b) comparable to that observed by Gerwig *et al.* [70], who prepared similar polystyrene sulfonate (PSS)/single-walled carbon nanotube (SWCNT)/PEDOT coatings. Typical fibrils possessed diameters over 70 nm greater than that of the MWCNTs, suggesting the presence of a uniform encapsulating film of PEDOT. Despite their delicate appearance, coated electrodes were able to be inserted and removed from cortical tissue with no visible change in appearance or impedance, indicating adequate mechanical resilience and adhesion to the substrate. The high surface roughness of the substrate apparent in Figure 1c, which resulted from the plasma arc method used to expose the electrode tips, may have had a positive effect on adhesion. The impact of substrate roughness on PEDOT stability was explored by Green *et al.* [88], who found that laser roughening of the substrate often resulted in significantly greater values of stimulation cycles-to-failure and CSC loss resistance of PEDOT coatings, though the benefit was observed to be dependent on the dopant employed.

Coated microelectrodes exhibited a significant decrease in 1 kHz impedance and an increase in charge storage capacity, which were observed by other studies using similar compositions [63,70,85]. This impedance decrease and charge storage capacity increase is a hallmark of conducting polymer coatings [51,89] and is the product of multiple factors including the high conductivity of the oxidized PEDOT and MWCNTs, the large capacitance provided by the enhanced surface area, and the charge transfer mechanisms available at the PEDOT interface due to redox activity and ion diffusion. The PEDOT/MWCNT/Dex coatings demonstrated a 1 kHz impedance decrease of ~40%, which was significant but substantially less than that observed by Gerwig *et al.* [70], who reported reductions of over 95% following PSS/SWCNT/PEDOT coating on gold MEAs. This contrast in performance is likely a consequence of the greater initial surface roughness of our Pt/Ir microwire tips compared to the planar gold MEA sites. Lower initial impedance would result in a decreased percentage from the same coating. In addition, dexamethasone is a poor dopant compared to PSS, which could have contributed to the increased resistance of the film.

### 4.2. In Vivo Cyclic Voltammetry Safety

In order to generate enough current to drive dexamethasone release without damaging the coating, a conservative electrochemical analysis paradigm was developed. The stimulus employed a cyclic voltammetry (CV) waveform which approximated charge balance while providing electrochemical feedback on both redox behavior and charge storage capacity. CV has been commonly employed to study conductive polymer properties, often as a stimulation for active drug release [48,90,91] though with much slower scan rates of 20–100 mV/s compared to the 1 V/s rate used here. These slow scan rates were deemed to be unfeasible *in vivo* due to the increased anesthesia time required as well as the likelihood of increased charge buildup and faradaic damage to the coating and tissue [49]. As mentioned in Section 3.3, a small surplus of anodic charge delivery was observed during charge storage capacity measurement. Also, with slow scan rates, the requirement that the stimulus pass below the −0.7 V (*vs.* Pt/Ir) PEDOT reduction potential could lead to a violation of the water window [3] and the possible evolution of hydrogen gas. Furthermore, the maximum safe stimulus voltage threshold is difficult to predict, due to the *in vivo* environment and the potential-controlled nature of stimulation. A fast CV scan rate minimizes the charge accumulation that may result in permanent damage of the electrode tissue interface. The continued presence of the PEDOT coating was verified by the reduction peak during CV stimulation in all coated channels throughout the experimental period. Material stability following CV was verified using SEM imaging of explants. SEMs showed that electrode dimensions and morphology remained visually unchanged after over 200 cycles. Interface integrity was monitored through post-CV impedance measurement. Neither the post-stimulus impedance nor gross anatomical histology revealed any obvious evidence of stimulation-induced lesion or gas evolution. Most importantly, the CV stimulus did not have any quantifiable impact on recording SNR, though a small but significant reduction in noise amplitude was observed from coated electrodes on the final day of implantation. CV scans also demonstrated consistent redox behavior and charge storage characteristics throughout the experiment. This evidence suggests that the majority of charge was transduced by way of safe, reversible mechanisms, and that the application of CV did not generate an observable degree of hydrolysis.

### 4.3. In Vivo Electrochemical Properties

The coated probes demonstrated dynamic multimodal changes in broad-spectrum impedance over the eleven day period of implantation, suggesting a progression of changes to physiological or material factors at the tissue/coating interface. This is contrasted with the behavior of uncoated probes, which exhibited a gradual increase in 1 kHz impedance typical of chronically implanted uncoated microelectrodes during the first week post-implantation in rat cortex [18,59,92]. This distinction between the sub-acute impedance behavior of PEDOT-coated and uncoated implanted electrodes was first noted by Abidian *et al.* [59] who observed complex changes to Nyquist representations of PEDOT nanotube-coated electrode sites which were not evident in uncoated controls, and coincided with a sharp increase in 1 kHz impedance during the initial 2-week period post-implantation. This increase in 1 kHz impedance has since been observed by others studying the *in vivo* performance of PEDOT-coated electrodes [53,66]. We observed similar phenomena in our Nyquist plots, suggesting that these changes to impedance behavior may be common to PEDOT-coated electrodes *in vivo*.

Nyquist plots collected from coated electrodes in PBS before implantation reveal the characteristic bimodal frequency response typically observed in conducting-polymer-coated electrodes, with low frequency behavior dominated by metal interface parameters *C_CPE_*, *R_CT_*, and β, and high frequency behavior characterized by an encapsulation element modeled using *Z_D_*. This is contrasted against Nyquist plots of uncoated electrodes in PBS, which in the measured frequency range (10 Hz to 32 kHz) demonstrate nearly linear constant-phase behavior characterized by *C_CPE_*, *R_CT_*, and β. The reduction in 1 kHz impedance between coated and uncoated probes *in vitro* most strongly correlates with *C_CPE_*, suggesting that the principal benefit of the coating is that of increasing the effective surface area of the double-layer capacitance. However, the coating also seems to contribute a diffusion barrier to the interface which is most apparent at high frequencies. It should be noted that within the frequency range measured, the ability of fitting techniques to distinguish between *R_CT_* and β is limited, particularly *in vivo* where substantial low frequency noise is often encountered during impedance measurement.

The gradual increase of average *in vivo* 1 kHz impedance of uncoated electrodes is typically characterized by subtle changes in *R_CT_* and β which dominate low-frequency behavior, as well as with a gradually emerging high-frequency diffusion barrier and encapsulation element modeled using *Z_D_*. These changes coincide with known physiological events thought to play a role in evolving electrode *in vivo* impedance, with *R_CT_* and β representing changes to electrode surface properties due to the protein adsorption that takes place immediately upon implantation, and *Z_D_* representing the growing boundary effect of inflammation, microglial encapsulation, and edema [17,26]. Chronic *in vivo* studies using uncoated microelectrodes have shown that 1 kHz impedance tends to peak at 9–15 days and then reduces to an intermediate magnitude where it typically remains at a fluctuating plateau. This is thought to correspond with the reduction of initial acute inflammation and tissue swelling, and the transformation of the interface to a stable chronic inflammatory state [18,59].

In contrast to the behavior of uncoated electrodes, the coated electrodes demonstrated an initial low-impedance period followed several days later by a rapid increase consistent between all coated electrodes. The initial low-impedance period persisted for between 3 to 4 days, with the nadir occurring between days 1 and 2. Fitted model parameters *C_CPE_* and *Q*_1_ correlate with this impedance low-point when averaged, suggesting that the coating required a one day “maturation” period following implantation to achieve its full benefit. The drop of impedance in the two days following implantation is possibly due to the time required for electrolyte to fully penetrate the pores of the coating, or for the fluid and tissue around the probes to stabilize post-implantation. Between day 3 and day 5 post-implantation, the average 1 kHz impedance of coated probes increased substantially to the point of equivalence with that of uncoated probes. Nyquist plots reveal that this increase is distinct from the increase observed in uncoated probes, and is principally due to large decreases in parameters *C_CPE_* and *Q*_1_, which allowed the encapsulation element *Z_D_* to dominate increasingly greater portions of the measured frequency range. These modeling results suggest that beginning at day 3–5, the surface area enhancement of the coating was sharply reduced and that a barrier composed of coating and inflammatory tissue elements began to dominate impedance behavior by reducing the exposed surface area, thereby reducing the capacitance of the conducting polymer. This hypothesis is supported by explant SEM imaging which revealed the presence of a dense membranous substance enveloping and interpenetrating the coating pores of all coated electrodes. Due to explant preparation for imaging, this substance was compromised before identification could be performed, but it is speculated to be a combination of cellular processes from fibroblasts, macrophage and microglia as well as dense extracellular matrix. Despite the impedance changes, the recording performance of the coated electrodes did not appear to be detrimentally affected, suggesting that the encapsulation element is limited to the area immediately surrounding and within the coating.

It should be noted that we employed the transmission-line linear diffusion element *Z_D_* to model both the encapsulation component of the conducting polymer coating as well as the ionic diffusion barrier of tissue encapsulation. *Z_D_* has been used to model each of these elements individually in different previously-published studies [84,85]. We speculate that in most circumstances it is unlikely that the impedance contribution of each can be confidently differentiated using measurement and circuit modeling alone, particularly if both coating and tissue encapsulation exhibit similar time constants. To better understand the impact of tissue encapsulation on electrode electrochemical behavior, a tissue decoupled *in vitro* chronic PBS soak test was performed using comparably-coated electrodes (see Section 4.4).

### 4.4. In Vitro Coating Evaluation

To decouple the impact of chronic soaking and repeated CV on the coated probes from the *in vivo* host tissue reaction, a number of electrodes were coated in a manner identical to those implanted *in vivo* and continuously soaked in PBS within a custom airtight chamber at ambient temperature. The goal of this experiment was to better understand whether the behavior observed *in vivo* was due to tissue response or some intrinsic change to the coating such as mechanical breakdown or loss of conductivity. As discussed in Section 4.3, the encapsulating effects of insulative coatings and inflammatory tissue can be difficult to differentiate using equivalent circuit analysis methods.

In the absence of daily CV, coated electrodes exhibited relatively unchanging electrical characteristics over the 11 day period of electrolyte exposure. This suggests that, at least within subacute time scales, coating conductivity was not noticeably impacted by passive degradation mechanisms such as swelling or dopant leach. However, among those electrodes subjected to daily CV, a portion underwent large changes in electrical behavior. As the CV applies a voltage beyond the redox potential of the conducting polymer, the polymer “switches” to a reduced state, causing a number of repercussions including the release of dopant molecules and exchange of dopant with negative ions in the solution, and changes to the mechanical properties and volume of the polymer [93,94]. The actuation effect generated by these changing mechanical properties has been previously exploited to “pump” a bioactive substance from conducting polymer tubules [47]. However, the stiffening and unstiffening of the coating and the volumetric change with each CV cycle could also act to dislodge the coating from the electrode surface, which could create the electrical changes observed in this experiment. Importantly, these changes were not universally observed, as two of the five CV-stimulated electrodes exhibited no large increase in 1 kHz impedance. It is possible that the susceptibility of the coating to this damage mechanism is dependent on the thickness of the coating, as the electrode showing the least degree of impedance change also had the largest charge storage capacity while those electrodes that showed great impedance change also had the lowest charge storage capacities. While identical coating parameters were applied to each electrode, a degree of discrepancy in the final coating thickness is expected based on large variations in surface area and roughness of the electrodes provided by the vendor.

While the 1 kHz impedance increases observed in three of the five CV-stimulated electrodes *in vitro* is superficially similar to the impedance increases observed in those coated electrodes implanted *in vivo*, there are also a number of clear differences. 1 kHz impedance increases were observed universally in all *in vivo* electrodes, while only three out of five electrodes showed similar increases *in vitro.* The complex impedance behaviors exhibited by the electrodes *in vivo* and *in vitro* were also very different. *In vitro*, Nyquist plots revealed the progressive loss of a high-frequency time constant, suggesting that the electrodes over time were approaching the behavior of bare metal. *In vivo*, the opposite was observed, where the high-frequency time constant element was observed to grow and eventually dominate the frequency response of the electrode, suggesting the development of insulative encapsulation. Finally, those electrodes *in vitro* exhibited large decreases in charge storage capacity, while the charge storage capacities of electrodes *in vivo* showed very little change. Together, these differences suggest that the increases in 1 kHz impedance *in vivo* and *in vitro* were due to very different mechanisms. One possibility is that the supporting presence of the tissue around the electrodes *in vivo* prevented the coating detachment observed *in vitro*, while inflammatory tissue generated an encapsulating sheath. Another contributing factor is that CV-induced decomposition and mechanical change in the polymer film is likely to be more pronounced in PBS, where ion and water transport in and out of the film is much easier and faster than in the brain tissue which is diffusion limited and where ions and water are not as readily available. Future studies that could explore chronic coating stability to greater detail include longer term soaking as well as age-accelerated [95] testing.

### 4.5. Neurophysiological Recording

In vivo neural recording was conducted to answer the following questions (1) will the PEDOT/MWCNT coating interfere with the recording capability; (2) will the CV stimulation cause any reversible or irreversible to the activity and health of the neurons nearby. Both spontaneous and evoked neural activity was recorded before and after daily CV and impedance measurement. A variety of different drifting solid grating visual stimulation programs were employed to drive neural activity, though for the purposes of this study all measures were averaged together across spontaneous and evoked blocks. In order to provide an assessment of raw recording performance, metrics of signal-to-noise ratio and noise amplitude were quantified. In general, only sparse unit activity was observed across both coated and uncoated probes over the initial week of implantation. However, both coated and uncoated probes exhibited well-defined units. Activity in both coated and uncoated probes increased substantially within recordings taken during the final days of implantation, with recording quality being essentially equivalent. This suggests that the multicomponent organic coating did not compromise the recording capability of the electrodes. Furthermore, we observed no different in SNR and yield immediately before and after the CV stimulation, indicating the stimuli applied here are mild enough to not cause any activity change. The inconsistent probe performance from day to day during the initial week post-implantation is likely due to the progression of acute inflammation and edema local to the implanted electrodes and the eventual stabilization of the interface tissue as it enters the chronic inflammatory stage, and is a rationale behind the common practice of delaying neural recording for the initial week post-implantation.

## 5. Conclusions

We demonstrate that the rapid sub-acute increases in the 1 kHz impedance of MWCNT-doped PEDOT coatings *in vivo* are due to the development of an encapsulation element, observed through analysis of the complex impedance behavior. We further demonstrate that this encapsulation element is not due to intrinsic changes within the coating itself, as comparable behavior was not observed in coated electrodes subjected to the same time period of electrolyte exposure and daily cyclic voltammetry *in vitro*. Despite the development of the encapsulating element, the coating was not observed to hinder neural recording and performed comparably with uncoated electrodes. This is in agreement with our longitudinal recording experiment with MWCNT-doped PEDOT sites [65]. Additionally, we observed no disturbance to neural activity upon CV stimulation. These findings set the stage for the long-term evaluation of electrically controlled drug release coatings for neural interface applications.
